# Factors Associated with Happiness among Malaysian Elderly

**DOI:** 10.3390/ijerph18073831

**Published:** 2021-04-06

**Authors:** Shamsul Azhar Shah, Nazarudin Safian, Saharuddin Ahmad, Wan Abdul Hannan Wan Ibadullah, Zulkefley bin Mohammad, Siti Rohani Nurumal, Juliana Mansor, Mohd Fairuz Addnan, Yugo Shobugawa

**Affiliations:** 1 Department of Community Health, Faculty of Medicine, Universiti Kebangsaan Malaysia, Cheras, Kuala Lumpur 56000, Malaysia; nazarudin@ppukm.ukm.edu.my (N.S.); p102417@siswa.ukm.edu.my (W.A.H.W.I.); p102416@siswa.ukm.edu.my (Z.b.M.); p102422@siswa.ukm.edu.my (S.R.N.); p102420@siswa.ukm.edu.my (J.M.); mfairuzaddnan@gmail.com (M.F.A.); 2 Department of Family Medicine, Faculty of Medicine, Universiti Kebangsaan Malaysia, Cheras, Kuala Lumpur 56000, Malaysia; saha@ppukm.ukm.edu.my; 3 Division of International Health (Public Health), Niigata University Graduate School of Medical and Dental Sciences, Niigata 950-2181, Japan; yugo@med.niigata-u.ac.jp; 4 Department of Active Ageing, Niigata University Graduate, School of Medical and Dental Sciences, Niigata 951-8510, Japan

**Keywords:** happiness, factor, elderly, Japan gerontological evaluation study

## Abstract

Happiness is an essential component to experience healthy ageing. Hence, understanding the factors that contribute to happiness is important. This study aimed to determine the factors associated with happiness among the elderly population in Malaysia. In this study, 1204 respondents were recruited from urban and rural areas in Selangor. A face-to-face interview was conducted using the Bahasa Malaysia version of the Japan Gerontological Evaluation Study questionnaire. The inclusion criteria include Malaysians who are 60-years old and above and can converse in the Malaysian language. Those who encounter less than seven scores for the Abbreviated Mental Test were excluded from the study. Among the 1204 respondents, 953 (79.2%) were happy. Sociodemographic characteristics showed that being a men, age of 60 to 74 years, and living in urban areas were significantly associated with happiness. A logistic regression model showed that locality (aOR 1.61), income category (Bottom 40% aOR 0.49; Middle-class group 40% aOR 1.40), social engagement (active aOR 1.77; less active aOR 1.25), receiving emotional support (aOR 2.11) and handgrip strength (aOR 1.02) were significantly associated with happiness. Thus, ensuring the elderly population in receiving emotional support and active social engagement among them can enhance their happiness level.

## 1. Introduction

As the population of the world is turning into an aged population, elderly health has become one of the priority aspects [1]. This demographic transition will have an impact on all aspects of society. By 2050, it is estimated that 20% of the world population will be 60-years or older, amounting to two billion people. The 2030 Agenda for Sustainable Development targeted healthy life for all; thus, everyone has the right to health regardless of their age [2]. Healthy life involves not only the physical health but also the mental and social well-being of the person, and is not only restricted to the presence of disease or infirmity [3] but includes also happiness.

Happiness is an essential factor for healthy ageing [4]. It is defined as a positive inner experience that results from an emotional interpretation of one’s lives and also the individuals’ cognitive function [5], or in short how a person likes the life he or she has [6]. The emotional component refers to pleasure (balance between comfort and pain or unpleasant effect), whereas the cognitive component is attributed to mental health [6]. The term happiness sometimes is interchangeable with life satisfaction. However, life satisfaction is mostly used for overall happiness. Life satisfaction related to happiness does not mean quality of life (QOL). The satisfaction in quality-of-life concept denotes life results’ inner qualities, which is the subjective QOL; yet, it does not represent life satisfaction. The concept of life satisfaction comprises the components of enduring and life-as-a-whole [6]. The term happiness is typically used for the affective appraisal of life, and it is synonymous with the hedonic level of effect [7]. Steptoe et al. divided subjective well-being into three components, namely ”affective well-being”, ”eudaimonic well-being” and ”evaluative well-being”.

Within this taxonomy, the positive feelings experienced by a person, such as happiness and joy, were classified as affective well-being. The eudaimonic well-being focuses on evaluations of meaning and purpose in life, including personal growth, positive relations with others and self-acceptance. Meanwhile, the evaluative well-being concept is related to the appraisals of how satisfied people are with their QOL [8]. Steptoe et al. have summarized eight factors that contribute to happiness, namely education, socioeconomic status, social network, time use and activities, stress exposure, marital status and family, personality and genetics [9]. There are many ways to assess happiness that depend on the type of subjective well-being components that they intend to measure and the time period considered. The responses can be either in verbal scales, numerical scales or graphical scales [6].

In Malaysia, study of happiness among the elderly population has not been well investigated. A study done in the year 2016 of the Malaysian adult population with an average age of 40 years noted that most of the respondents (96%) were happy [10]. Happiness was significantly associated with high household income, employment, high education level and being female. However, it was not significantly associated with age. Meanwhile, a study by Park et al. [11] showed that happiness declined with age in Malaysia and was especially low for those who were 50 years and older. Previous studies on life satisfaction have been performed; however, in view of the period of the study, the fast-growing numbers of elderly people in Malaysia and current changes in the socioeconomic surrounding, we believe it is necessary for us to have a more updated study in order to plan for proper elderly policy to be implemented.

Hence, this study aimed to determine the overall happiness and its associated factors among the elderly population in Malaysia, focusing on social engagement and support, education, socioeconomic status, time use and activities, depression status, marital status and family.

## 2. Materials and Methods

### 2.1. Design and Population of the Study

A cross-sectional study was conducted among older adults aged 60-years and above in four areas in Selangor, Malaysia from 1 December 2018 to 30 April 2020. Selangor is a state that surrounds the capital city of Kuala Lumpur on the west coast of Peninsular Malaysia. Selangor is the most populous state in the country with 6.53 million inhabitants in 2020 [12]. It reflects the nation’s diversity of people and living conditions and includes all major ethnic groups, such as Malays, Chinese people and Indians.

The method used in this study was similar to that of Safian et al. [13], whereby the official public data on district administrative units and their population were used as sampling frame. There are nine districts and 177 sub-districts in Selangor. Hulu Langat district, with more than one million inhabitants, was picked as a representative of urban regions. Meanwhile, Kuala Selangor, with 0.2 million inhabitants, was chosen as a representative of rural regions. Multi-stage cluster sampling was performed, with a probability proportionate to the size of the elderly population. Districts, specifically Hulu Langat and Kuala Selangor, are the primary sampling units, whereas sub-districts are the secondary sampling units. Six sub-districts were chosen, respectively, from Hulu Langat (seven sub-districts) and Kuala Selangor (nine sub-districts). Ten towns/villages were randomly chosen from each sub-district at the third sampling point. There are around 30–50 towns/villages in a typical sub-district. With permission from the appropriate village head, the household ledgers for the selected areas were obtained and used as the sampling system for households and individuals. A random sampling of households with an older individual from the areas selected was carried out. The Kish grid table was used to pick the sample when more than one older adult in a chosen household was eligible for the analysis. The sample size was determined using the equation n = Z2[(P(1 − P))/e2)] [14], where Z is the confidence level, P is the prevalence of ‘good health’ among older people, and e is the error margin. Using Z = 1.96, P = 0.3 (estimate from a previous analysis of older individuals in Japan) [15] and e = 0.05, the initial sample size measurement was 322. The design effect of 1.5 and the two groups of estimates (urban and rural) needed for the survey results = 966 were then multiplied by this initial sample size. Finally, 966 was divided by 0.80 to account for an estimated 20% non-response rate, resulting in a total sample size of 1207. Finally, with a response rate of 99.8%, we successfully recruited 1204 respondents.

Before the interview, respondents were given a detailed description of the study, including information sheets and consent forms. The interviews were conducted in a private environment, face-to-face, by professional research assistants immediately after the respondents signed the consent document and lasted 40–50 min. The study used the Bahasa Malaysia variant of the questionnaire for the Japan Gerontological Assessment Study (BM-Japan Gerontological Evaluation Study (JAGES)) [16], which incorporates multi-dimensional variables for healthy ageing. The inclusion criteria for respondents were (1) being at least 60-years of age and able to speak the Malaysian language; (2) being registered residents of Malaysia (as the sampling frame was used for household ledgers); (3) living at home and (4) being able to understand and consent to comply with the study. However, if the respondent was unable to cooperate and had an Abbreviated Mental Test score of less than seven in the screening questions, the individual was excluded. Those who were institutionalized in nursing or old folks’ homes were also excluded. The study has been approved by the Research Ethics Committee of the National University of Malaysia (FF-2018-532).

### 2.2. Variables

The self-perceived happiness was subjectively determined by the respondents using a single question with a scale from 0 to 10 points. In this study, happiness refers to the overall happiness in life. The question was as follows: To what degree do you feel you are currently happy? (Score ”0” for ”Very unhappy” and ”10” for ”Very happy”). Based on this scale, happiness was defined as a self-rated score of 7–10 points, whereas unhappiness 0–6 points. This level of the cut points was finalized using a previous survey among the elderly in Japan on their average happiness score, which was six points [11]. This single question was used to measure happiness in a previous article in Japan using the JAGES questionnaire [17].

Sociodemographic variables included age, sex, marital status and locality. Age was categorized into the following three levels: 60–74, 75–84 and ≥85 years. On the basis of the article by Steptoe et al., we were able to include five components, namely marital status, socioeconomic status, education, social network and physical activities. Marital status was categorized into married living together, married living separately, widowed and divorced and never married. Locality was divided into rural and urban.

Socioeconomic status includes household income, education level and current employment status. For household income classification, we used the Income Structure 2019 by the Department of Statistics Malaysia [18]. B40 refers to the base group or bottom 40%, with a monthly household income of less than RM4850, whereas M40 refers to the middle-class group or middle 40%, with earnings between RM4851 and RM10,959 per month. Conversely, T20 is the upper-class group or top 20%, with monthly earnings of more than RM10959 [12].

In the study, we used social engagement and social support in measuring social network. Social network was measured with the frequency of engagement in group activities. The activities that were measured were religious, volunteering, sports or clubs, hobbies, community meetings and political meetings or events. Participation at least once a week in any one of these activities was considered active engagement. Meanwhile, social support was measured by asking respondents if they have someone to talk to regarding concerns or complaints (emotional support) and who looked after them when they felt sick and were confined for a few days (instrumental support). These variables were also used by Park et al. in defining social support [10]. We also asked if they listen to someone’s concerns or complaints and whether they look after someone when he/she is sick and confined for a few days.

Physical measurements comprised the body mass index (BMI) and handgrip strength (HGS). Weight and height were measured twice to calculate the BMI. The Malaysian BMI classification was used as a reference [13], in which underweight is defined as BMI < 18.5 kg/m^2^, normal weight as BMI = 18.5–22.9 kg/m^2^, overweight as BMI ≥ 23 kg/m^2^, pre-obese as BMI = 23.0–27.4 kg/m^2^, obese I as BMI = 27.5–34.9 kg/m^2^, obese II as BMI = 35.0–39.9 kg/m^2^ and obese III as BMI ≥ 40 kg/m^2^ [19]. HGS was measured using a handgrip dynamometer with the dominant hand (T.K.K. 5001 GRIP-A; Takei Scientific Instrument Co. Ltd., Japan). The HGS of each of the respondents was measured twice, and the mean was taken as reading.

Physical activities for older adults were evaluated through questions regarding the frequency of moderate exertion activities such as brisk walking, golf, dancing, farming, gardening or car washing. This later was translated to an average of 150 min of moderate-intensity aerobic physical activity throughout the week based on the WHO recommendation [20].

Respondents were asked about their diseases or comorbidities. These include stroke, heart disease, diabetes, hyperlipidaemia, respiratory disease, gastrointestinal, liver or gallbladder disease, kidney or prostate gland disease, musculoskeletal disease, including osteoporosis and arthritis, traumatic injury, cancer, blood or immune disease, depression, dementia, Parkinson’s disease, eye and ear disease, malaria and HIV infection and gynecological problem.

### 2.3. Data Analyses

To determine the associations among the study variables, chi-squared and independent *t*-tests were conducted. Simple logistic and multiple logistic regressions were used to calculate the crude and adjusted odds ratios, respectively. A *p*-value of <0.003 (Bonferroni correction) was considered significant. Any variables from the simple logistic regression with a *p*-value of <0.25 were candidates for the multivariable model [21]. Once the multivariable analysis was completed, the preliminary model was checked for multicollinearity and interactions. Analyses were conducted using IBM SPSS version 21.0 (IBM Corp., Armonk, NY, USA).

## 3. Results

Among 1204 respondents, 953 (79.2%) reported to be happy. Of these, 57.4% were male, the majority (82.7%) was 60 to 74 years old, 65.6% was married and 94.2% stayed with blood-related family members (Table 1).

### 3.1. Association of Happiness with Sociodemographic Factors

Happiness was significantly associated with sociodemographic factors such as age, sex and locality, as shown in Table 1. Individuals of the younger age group (60–74 years) were happier than those in the other two groups (*p* = 0.031). In terms of sex, being male was significantly associated with happiness compared with being female (*p* = 0.031). In terms of the location of residence, the elderly who lived in urban places were happier in their daily life (*p* < 0.001).

### 3.2. Association of Happiness with Socioeconomic Status

The household income was categorized into three main groups in this study; the respondents in the M40 group showed to be significantly happier than those in the other two groups. In the same group, those who were employed considered themselves happier than those in the other two groups, although the difference was not statistically significant.

### 3.3. Association of Happiness with Health Parameters

Happiness was significantly associated with health parameters such as moderate physical activity, comorbidities and HGS as shown in Table 2. BMI was not significantly associated with happiness. In terms of presence of chronic diseases or comorbidities, those who did not have comorbidities were significantly happier (*p* = 0.042). Meanwhile, for HGS measurement, those who were happy demonstrated higher HGS (mean = 26.0 kg, *SD* = 8.61) than those who were unhappy.

### 3.4. Association of Happiness with Social Network

Most of the respondents had engaged in social group activities as shown in Table 3. Only 17.9% of them were not involved in any kind of group activity. Frequent engagement in social activity was significantly associated with happiness (*p* ≤ 0.001). The majority of the participants (60.6%) engaged in religious group activities, followed by community meetings (9.6%), hobbies (5.8%), political meetings or events (2.6%), sports or clubs (2.0%) and volunteer group activities (1.6%).

Most of the respondents received and acted as providers of social support. However, having someone to talk to regarding their concerns and problems (*p* = 0.002) and someone to look after them when sick (*p* = 0.031) were significantly associated with happiness. The main person who was accountable for providing emotional support to the respondents was their spouse (50.2%). Conversely, the main persons that provide instrumental support to the respondents were their children who are living with them (56.5%).

In the multivariable analysis, there were five factors associated with happiness as follows: location, comorbidities, social engagement, receiving emotional support and HGS (Table 4). The model correctly classified 79.1% of the respondents. Neither interaction nor collinearity was present.

In terms of locality, those living in urban areas had 61% higher odds of being happy compared with those in rural areas. Those reported to have no comorbidities had 46% higher odds of being happy compared with those suffering from comorbidities. Those receiving emotional support were twice more likely to be happy compared with those not receiving any emotional support.

## 4. Discussion

The findings of this study showed that 79.2% of the elderly who participated in the study were happy and the factors that contributed to their happiness were significantly associated with their locality, comorbidities, social engagement with the community, HGS and receiving any emotional support. Comparatively, the BM-JAGES conducted in Japan involving Japanese older adults aged 65 years and above showed that 68% of the participants self-rated themselves to have overall happiness. [17]. The percentage of overall happiness in Japan is slightly lower than that in Malaysian elderly based on our study. With greater life expectancy in both men and women in Japan, the elderly are prone to loneliness and social isolation. The famous Japanese word “*kodokushi*”, which refers to dying alone with the corpse that remained undiscovered for a long period of time, commonly makes Japanese elderly anxious as they age [22]. This might contribute to the lower percentage of overall happiness among Japanese elderly compared with the Malaysian elderly. Peer-based intervention as one of the methods to overcome loneliness and social isolation among the elderly has been shown to enhance happiness among them [23].

In terms of locality, the elderly living in urban areas are happier. This is similar to the finding in a previous study conducted among elderly of age 60-years old and above at Chonburi Province, Thailand, as they can have access to some hobbies or activities. The elderly living in urban areas receive regular monthly retirement pension, whereas those in rural areas still have to work at an advanced age as they only have subsistence allowance [24].

Multivariable analysis showed that there is no significant association between household income and happiness. A similar finding was found in a study done in Brazil involving 236 people [25]. In general, a person with a higher income is happier than one with a lower income. Having enough money will allow one to fulfil his/her material needs, thus affecting his/her happiness. A study done among Turkish elderly showed that low-income levels increased the odds of being unhappy by four times [26]. By contrast, having an income beyond one’s needs does not affect happiness [27]. The low socioeconomic status among older people is detrimental and directly affects their mental health and subsequently increase the risk for suicidal ideation [28]. A Korean national-level study noted that household income is associated with interpersonal trust and depressive symptoms [29,30]. In this study, the less depressive a person is the happier he/she is. A study noted that adjusting for depression and pension types has an effect on the happiness status [17].

Considering receiving support, older people receiving emotional support showed a more positive view of happiness, and this was similar to that shown in studies in Iran and Japan [31,32]. In 2018, a study was conducted in Thailand to determine the factors that affect the QOL among the elderly population; it showed that emotional support did contribute significantly to improving the QOL of elderly people [33]. Emotional loneliness among the elderly has been shown to be associated with increased risk of all-cause mortality. Emotional loneliness or not receiving any emotional support happens due to loss or absence of a close emotional attachment figure that can provide a sense of belonging, of companionship, and of being a member of the community [34].

A study done in the United States showed that a community-based intervention for elderly individuals consisting of 90-min group sessions involving elderly receiving emotional support in terms of having positive relationship among others led to a significant increase in life satisfaction and happiness and lowered the levels of depression among the participants [35]. This showed that emotional support among the elderly is crucial to achieve higher levels of happiness leading to healthy ageing. Having family and friends as support has been shown to lead to a better level of overall happiness over a stable period during the ageing process [36].

This study also found that not having any comorbidities significantly contributed to happiness among the elderly. A cross-sectional study conducted among elderly Nepalese showed a similar result, i.e., that elderly individuals without depression based on a geriatric depression scale were happier [37]. A similar finding in Iran showed elderly individuals diagnosed with hypertension had lower happiness levels compared with those without hypertension [38]. A similar result was obtained in one of the studies conducted among older Korean women living alone to determine the predictors of their happiness. The study found that happiness was negatively correlated with the number of comorbidities and having depressive symptoms [39]. Interestingly, a study in Brazil among people with end-stage renal failure undergoing hemodialysis showed that even though they suffered from chronic kidney diseases, a high level of religiosity led to higher happier [40].

Based on the result, having active social engagement is significantly associated with happiness among the elderly. Social engagement or participation among elderly individuals refers to community-based activities and interpersonal interactions, revolving around resource sharing, active participation and individual satisfaction. It has the elements of individual, environmental and social background, as well as implications on the individual and environment [41]. Individuals with low social participation were found to have a steep decline in psychological well-being, which was not buffered by social support [42]. A similar finding was noted among Chinese elderly whereby social participation had a profound effect on life satisfaction and depression compared with social support [43]. Moreover, individuals with high social participation tend to have more social support than those with low participation [43] since they actively interact and mingle around. Hence, having active social participation was more crucial than having social support [42,43] in determining happiness status among the elderly.

In term of HGS, the higher the mean score associated with happiness among the elderly. This is an anthropometric measurement that is also known as arm strength that specify the health of the arm muscle. The HGS also as the indicators of the elderly well- being [44]. Thus, it includes the fundamental of their QOL as well the happiness in daily living [45]. In consequence the HGS, can establish happiness among the elderly population following healthy life style such as being physically active [46]. This was also shown in a study conducted in the younger generation of 145 University students in British, which showed that HGS correlated significantly (r = 0.43) with happiness as the participants perceived themselves on the emotion [47]. Hence, HGS can either directly or indirectly determine the happiness among the population, the young as well the elderly.

This study has its own limitations. The older people are not a homogeneous group of frail individuals who progress rapidly towards disease and need of care. Successful and healthy ageing can also be determined by their personality traits, which were not explored in this study. According to Kahlbaugh et al., to have a successful ageing, being open and teachable and having low neuroticism are important factors [48]. Therefore, the personality traits of each elderly individual are additional factors that might contribute to their happiness level. Second, the overall happiness level measure among the respondents was based on a single-item questionnaire and it is a self-rated questionnaire. No confirmatory questions followed. Nonetheless, this method of assessing overall happiness has been commonly used in previous published studies and it is moderately reliable [49,50,51]. This is the limitation in the JAGES questionnaire. Thus, in the future, we plan to conduct more studies on the life style/well-being and QOL domains among the elderly population in Malaysia.

When considering who shall provide emotional support for the elderly, family members are the first to be thought of. Hence, more awareness programs must be initiated among family members regarding emotional support for their elderly. However, there are several circumstances in which family members cannot provide support; hence, having various sources of support is essential. Community-based services are useful for the elderly especially for those who are living alone. Support for the elderly can be found in several places including assisted living facilities, homes or care centers for the elderly, meal delivery or even religious affiliations. These services can provide positive support, either emotional or instrumental, which can help the elderly defeat loneliness and isolation.

## 5. Conclusions

To conclude, for the benefit of the next generation of older people, emotional support and active social engagement among them should be assured to promote lifelong happiness. This study has shown some determinants that we need to look at and explore further to achieve happiness among the Malaysian elderly. Therefore, programs or activities should be structured and established with the aim of strengthening the emotional support and active social engagement in the elderly population.

## Figures and Tables

**Table 1:** Descriptive and bivariate analysis of factors associated with happiness.

Variables	N	(%)	Happiness, N (%)		^a^*p*-Value
			Happy	Unhappy	
Age group					
Young old (60–74 years)	996	82.7	801 (80.4)	195 (19.6)	0.031
Middle old (75–84 years)	186	15.4	138 (74.2)	48 (25.8)	
Old old (≥85 years)	22	1.8	14 (63.6)	8 (36.4)	
Sex					
Male	691	57.4	562 (81.3)	129 (18.7)	0.031
Female	513	42.6	391 (76.2)	122 (23.8)	
Marital status					
Married	790	65.6	653 (81.4)	1469(18.6)	0.023
Widowed or divorced	384	1.8	287 (74.7)	97 (25.3)	
Never married	18	1.5	13 (72.2)	5 (27.8)	
Household composition					
Stay alone	64	5.3	54 (84.4)	10 (15.6)	0.253
Stay with blood-related family	1134	94.2	893 (78.7)	241 (21.3)	
Stay with other family (non-blood-related)	6	0.5	6 (100.0)	0 (0)	
Locality					
Rural	602	50.0	450 (74.8)	152 (25.2)	<0.001
Urban	602	50.0	503 (83.6)	99 (16.4)	
Education level					
Primary or less	665	55.2	513 (77.1)	152 (22.9)	0.035
Secondary	426	35.4	341 (80.0)	85 (20.0)	
Tertiary	113	9.4	99 (87.6)	14 (12.4)	
Current employment status					
Employed	169	14.0	139 (82.2)	30 (17.8)	0.560
Retired from job	868	72.1	682 (78.6)	186 (21.4)	
Never had a job	167	13.9	132 (79.0)	35 (21.0)	
Household income					
B40	1094	90.9	851 (77.8)	243 (22.2)	0.001
M40	98	8.1	91 (92.9)	7 (7.1)	
T20	12	1.0	11 (91.7)	1 (8.3)	

^a^ Chi-squared test, *p*-value < 0.003 considered significant.

**Table 2:** Descriptive and bivariate analysis of health parameters associated with happiness.

Variables	N	(%)	Happiness, N (%)		^a^*p*-Value
			Happy	Unhappy	
Physical activity					
Yes	632	52.5	516 (81.6)	116 (18.4)	0.025
No	572	47.5	437 (76.4)	135 (23.6)	
BMI class					
Normal	223	18.5	167 (74.9)	56 (25.1)	0.145
Obese I	410	34.1	329 (80.2)	81 (19.8)	
Obese II	61	5.1	44 (72.1)	17 (27.9)	
Obese III	17	1.4	15 (88.2)	2 (11.8)	
Pre-obese	454	37.7	370 (81.5)	84 (18.5)	
Underweight	39	3.2	28 (71.8)	11 (28.2)	
Comorbidity					
Yes	941	78.2	733 (77.9)	208 (22.1)	0.042
No	263	21.8	220 (83.7)	45 (16.3)	
Handgrip strength					
Mean (SD) in kilograms			26.0 (8.61)	23.5 (7.89)	<^b^ 0.001

^a^ Chi-squared test, ^b^ Student *t*-test.

**Table 3:** Descriptive and bivariate analysis of social network associated with happiness.

Variables	N	(%)	Happiness, N (%)		^a^*p*-Value
			Happy	Unhappy	
Social engagement					
Active	687	57.1	574 (83.6)	113 (16.4)	<0.001
Less active	301	25.0	230 (76.4)	71 (23.6)	
Never	216	17.9	149 (69.0)	67 (31.0)	
Receiving social support					
Emotional					
Yes	1126	93.5	902 (80.1)	224 (19.9)	0.002
No	78	6.5	51 (65.4)	27 (34.6)	
Instrumental					
Yes	1117	92.8	892 (79.9)	225 (20.1)	0.031
No	87	7.2	61 (70.1)	26 (26.9)	
Providing social support					
Emotional					
Yes	1080	89.7	860 (79.6)	220 (20.4)	0.229
No	124	10.3	93 (75.0)	31 (25.0)	
Instrumental					
Yes	982	81.6	783 (79.7)	199 (20.3)	0.295
No	222	18.4	170 (76.6)	52 (23.4)	

^a^ Chi-squared test.

**Table 4:** Factors associated with happiness.

Variables		Crude OR (95% CI)	^d^*p*-Value	Adj OR (95% CI)	^d^*p*-Value
Locality					
	Urban	1.72 (1.29, 2.28)	<0.001	1.61 (1.21, 2.16)	0.001
	Rural	1.00		1.00	
Income category					
	B40	0.32 (0.04, 2.48)	^c^ 0.274	0.49 (0.62, 3.95)	^c^ 0.506
	M40	1.18 (0.13, 10.52)	^c^ 0.881	1.40 (0.15, 12.72)	^c^ 0.767
	T20	1.00		1.00	
Comorbidities					
	No	1.45 (1.01, 2.08)	0.043	1.46 (1.01, 2.11)	0.047
	Yes	1.00		1.00	
Social engagement					
	Active	2.28 (1.61, 3.25)	<^c^ 0.001	1.77 (1.21, 2.59)	^c^ 0.003
	Less Active	1.46 (0.98, 2.16)	^c^ 0.060	1.25 (0.83, 1.87)	^c^ 0.283
	Never	1.00		1.00	
					
Receiving emotional support					
	Yes	2.13 (1.31, 3.48)	0.002	2.11 (1.28, 3.50)	0.004
	No	1.00		1.00	
					
Handgrip Strength		1.04 (1.02, 1.05)	<0.001	1.02 (1.00, 1.04)	0.017

^c^ Wald test; ^d^ likelihood ratio test. Model adjusted for age group, sex, marital status, household composition, education level, current employment status, physical activity, BMI, receiving instrumental social support and providing emotional and instrumental social support.

## Data Availability

The research datasets analysed during the current study are available in the Mendeley dataset repository. [https://data.mendeley.com/datasets/t5nb6g859m/1] (accessed on 9 February 2021).

## References

[1] Strategy W.G. (2017). Action Plan on Ageing and Health.

[2] Nations U. (2015). Transforming our World: The 2030 Agenda for Sustainable Development.

[3] (1946). Constitution of the World Health Organization. Am. J. Public Health Nations Health.

[4] Bum C.H., Johnson J.A., Choi C. (2020). Healthy aging and happiness in the Korean elderly based upon leisure activity type. Iran. J. Public Health.

[5] Diener E., Oishi S., Lucas R.E. (2003). Personality, culture, and subjective well-being: Emotional and cognitive evaluations of life. Annu. Rev. Psychol..

[6] Cohen S., Herbert T.B. (1996). Health psychology: Psychological factors and physical disease from the perspective of human psychoneuroimmunology. Annu. Rev. Psychol..

[7] Veenhoven R. (2012). Happiness: Also known as ‘life-satisfaction’ and ‘subjective well-being’. Handbook of Social Indicators and Quality of Life Research.

[8] Steptoe A., Deaton A., Stone A.A. (2015). Subjective wellbeing, health, and ageing. Lancet.

[9] Steptoe A. (2019). Happiness and health. Annu. Rev. Public Health.

[10] Boo M.C., Yen S.H., Lim H.E. (2016). A note on happiness and life satisfaction in Malaysia. Malays. J. Econ. Stud..

[11] Park M.S.A., Joshanloo M. (2019). Satisfaction with life declines with age in Malaysia: An exploratory analysis of factors influencing subjective well-being in a developing/middle-income country. Appl. Res. Qual. Life.

[12] Department of Statistics Malaysia (2015). Selangor. https://www.dosm.gov.my/v1/index.php?r=column/cone&menu_id=eGUyTm9RcEVZSllmYW45dmpnZHh4dz09.

[13] Safian N., Shah S.A., Mansor J., Mohammad Z., Nurumal S.R., Ibadullah W.A.H.W., Ahmad S., Shobugawa Y. (2021). Factors associated with the need for assistance among the elderly in Malaysia. Int. J. Environ. Res. Public Health.

[14] World Health Organization (2005). WHO STEPS Surveillance Manual: The WHO STEPwise Approach to Chronic Disease Risk Factor Surveillance/Noncommunicable Diseases and Mental Health.

[15] Nishi A., Kondo K., Hirai H., Kawachi I. (2011). Cohort profile: The AGES 2003 cohort study in Aichi. Japan. J. Epidemiol..

[16] Kondo K. (2016). Progress in aging epidemiology in Japan: The JAGES project. J. Epidemiol..

[17] Sasaki I., Kondo K., Kondo N., Aida J., Ichikawa H., Kusumi T., Sueishi N., Imanaka Y. (2018). Are pension types associated with happiness in Japanese older people? JAGES cross-sectional study. PLoS ONE.

[18] Department of Statistics Malaysia Pocket Stats Quarter 4. https://www.dosm.gov.my/v1/uploads/files/7_Publication/Infographic/PocketStats/Q4-2019/Pocket_Stats_Q4-2019.pdf.

[19] Alamuddin N., Bakizada Z., Wadden T.A. (2016). Management of obesity. J. Clin. Oncol..

[20] World Health Organization (2010). Global Recommendations on Physical Activity for Health: 65 Years and Above.

[21] Hosmer D.W., Lemeshow S., Sturdivant R.X. (2000). Applied Logistic Regression.

[22] Suzuki K., Dollery B.E., Kortt M.A. (2020). Addressing loneliness and social isolation amongst elderly people through local co-production in Japan. Soc. Policy Adm..

[23] Lai D.W.L., Li J., Ou X., Li C.Y.P. (2020). Effectiveness of a peer-based intervention on loneliness and social isolation of older Chinese immigrants in Canada: A randomized controlled trial. BMC Geriatr..

[24] Sumngern C., Azeredo Z., Subgranon R., Sungvorawongphana N., Matos E. (2010). Happiness among the elderly in communities: A study in senior clubs of Chonburi Province, Thailand. Jpn. J. Nurs. Sci..

[25] Luchesi B.M., de Oliveira N.A., de Morais D., de Paula Pessoa R.M., Pavarini S.C.I., Chagas M.H.N. (2018). Factors associated with happiness in the elderly persons living in the community. Arch. Gerontol. Geriatr..

[26] Ergin I., Mandiracioglu A. (2015). Demographic and socioeconomic inequalities for self-rated health and happiness in elderly: The situation for Turkey regarding World Values Survey between 1990 and 2013. Arch. Gerontol. Geriatr..

[27] Moeini B., Barati M., Farhadian M., Babamiri M., Heydari Ara M. (2016). Happiness and its related factors among the elderly in Hamadan (Iran): A Cross Sectional Study. Avicenna J. Neuro Psycho Physiol..

[28] Ju Y.J., Park E.C., Han K.T., Choi J.W., Kim J.L., Cho K.H., Park S. (2016). Low socioeconomic status and suicidal ideation among elderly individuals. Int. Psychogeriatr..

[29] Han K.M., Han C., Shin C., Jee H.J., An H., Yoon H.K., Ko Y.H., Kim S.H. (2018). Social capital, socioeconomic status, and depression in community-living elderly. J. Psychiatr. Res..

[30] Song A., Kim W. (2020). The association between relative income and depressive symptoms in adults: Findings from a nationwide survey in Korea. J. Affect. Disord..

[31] Higuchi M., Suzuki K., Ashida T., Kondo N., Kondo K. (2018). Social support and access to health care among older people in japan: Japan gerontological evaluation study (JAGES). Asia Pac. J. Public Health.

[32] Moeini B., Barati M., Farhadian M., Ara M.H. (2018). The association between social support and happiness among elderly in Iran. Korean J. Fam. Med..

[33] Whangmahaporn P., Simmonds P., Whangmahaporn B. (2018). Factors affecting quality of life of the elderly in Thailand. SSRN Electron. J..

[34] O’Súilleabháin P.S., Gallagher S., Steptoe A. (2019). Loneliness, living alone, and all-cause mortality: The role of emotional and social loneliness in the elderly during 19 years of follow-up. Psychosom. Med..

[35] Friedman E.M., Ruini C., Foy R., Jaros L., Sampson H., Ryff C.D. (2017). Lighten UP! A community-based group intervention to promote psychological well-being in older adults. Aging Ment. Health.

[36] Lelkes O. (2008). Happiness Across the Life Cycle: Exploring Age-Specific Preferences.

[37] Ghimire S., Baral B.K., Karmacharya I., Callahan K., Mishra S.R. (2018). Life satisfaction among elderly patients in Nepal: Associations with nutritional and mental well-being. Health Qual. Life Outcomes.

[38] Fouladivanda S., Zibaeenezhad M.J., Moghimi E., Razeghian-Jahromi I. (2018). Investigating the effect of hypertension on happiness and quality of life in a population from Shiraz. Int. Cardiovasc. Res. J..

[39] Kim J., Song Y., Kim T., Park K. (2019). Predictors of happiness among older Korean women living alone. Geriatr. Gerontol. Int..

[40] Siqueira J., Fernandes N.M., Moreira-Almeida A. (2019). Association between religiosity and happiness in patients with chronic kidney disease on hemodialysis. J. Bras. Nefrol..

[41] Aroogh M.D., Shahboulaghi F.M. (2020). Social participation of older adults. Int. J. Commun. Based Nurs. Midwif..

[42] Sharifian N., Grühn D. (2019). The differential impact of social participation and social support on psychological well-being: Evidence from the Wisconsin longitudinal study. Int. J. Aging Hum. Dev..

[43] Li C., Jiang S., Li N., Zhang Q. (2018). Influence of social participation on life satisfaction and depression among Chinese elderly: Social support as a mediator. J. Commun. Psychol..

[44] Musalek C., Kirchengast S. (2017). Grip strength as an indicator of health-related quality of life in old age-a pilot study. Int. J. Environ. Res. Public Health.

[45] Teixeira L.E.P.P., Silva K.N.G., Imoto A.M., Teixeira T.J.P., Kayo A.H., Montenegro-Rodrigues R., Peccin M.S., Trevisani V.F.M. (2010). Progressive load training for the quadriceps muscle associated with proprioception exercises for the prevention of falls in postmenopausal women with osteoporosis: A randomized controlled trial. Osteoporos. Int..

[46] Bilajac L., Juraga D., Zuljevic H. (2019). The influence of physical Activity on handgrip strength of elderly. Arch. Gerontol. Geriatr. Res..

[47] Sneade M., Furnham A. (2016). Hand grip strength and self-perceptions of physical attractiveness and psychological well-being. Evol. Psychol. Sci..

[48] Kahlbaugh P., Huffman L. (2017). Personality, emotional qualities of leisure, and subjective well-being in the elderly. Int. J. Aging Hum. Dev..

[49] Veenhoven R. (2017). Measures of happiness: Which to choose?. Metrics Well-Being Limits Improve.

[50] Krueger A.B., Schkade D.A. (2008). The reliability of subjective well-being measures. J. Public Econ..

[51] Diener E., Wirtz D., Tov W., Kim-Prieto C., Choi D., Oishi S., Biswas-Diener R. (2010). New well-being measures: Short scales to assess flourishing and positive and negative feelings. Soc. Indic. Res..

